# Perceptions and Use of Telehealth Among Diverse Communities: Multisite Community-Engaged Mixed Methods Study

**DOI:** 10.2196/44242

**Published:** 2023-03-28

**Authors:** Amelia Barwise, Todd Huschka, Cecelia Woo, Jason Egginton, Lily Huang, Jay-Sheree Allen, Matthew Johnson, Kathryn Hamm, Wendy Wolfersteig, Sean Phelan, Megan Allyse

**Affiliations:** 1 Program in Biomedical Ethics Research Mayo Clinic Rochester, MN United States; 2 Division of Pulmonary and Critical Care Medicine Mayo Clinic Rochester, MN United States; 3 Robert D and Patricia E Kern Center for Science of Health Care Delivery Mayo Clinic Rochester, MN United States; 4 Davidson College Davidson, NC United States; 5 Quantitative Health Sciences Mayo Clinic Jacksonville, FL United States; 6 Department of Family Medicine Rochester, MN United States; 7 Department of Medicine Mayo Clinic Rochester, MN United States; 8 Office of Evaluation and Partner Contracts Southwest Interdisciplinary Research Center Arizona State University Phoenix, AZ United States; 9 Division of Health Care Delivery Research Mayo Clinic Rochester, MN United States; 10 Department of Obstetrics & Gynecology Mayo Clinic Rochester, MN United States

**Keywords:** telehealth, telemedicine, digital health, community-based research, mixed methods research, COVID-19, health care system, community health, health delivery, patient experience, quality of care

## Abstract

**Background:**

Telehealth has been increasingly adopted by health care systems since the start of the COVID-19 pandemic. Although telehealth may provide convenience for patients and clinicians, there are several barriers to accessing it and using it effectively to provide high-quality patient care.

**Objective:**

This study was part of a larger multisite community-engaged study conducted to understand the impact of COVID-19 on diverse communities. The work described here explored the perceptions of and experience with telehealth use among diverse and underserved community members during COVID-19.

**Methods:**

We used mixed methods across three regions in the United States (Midwest, Arizona, and Florida) from January to November 2021. We promoted our study through social media and community partnerships, disseminating flyers in English and Spanish. We developed a moderator guide and conducted focus groups in English and Spanish, mostly using a videoconferencing platform. Participants were placed in focus groups with others who shared similar demographic attributes and geographic location. Focus groups were audio-recorded and transcribed. We analyzed our qualitative data using the framework analytic approach. We developed our broader survey using validated scales and with input from community and scientific leaders, which was then distributed through social media in both English and Spanish. We included a previously published questionnaire that had been used to assess perceptions about telehealth among patients with HIV. We analyzed our quantitative data using SAS software and standard statistical approaches. We examined the effect of region, age, ethnicity/race, and education on the use and perceptions of telehealth.

**Results:**

We included data from 47 focus groups. Owing to our mode of dissemination, we were not able to calculate a response rate for the survey. However, we received 3447 English-language and 146 Spanish-language responses. Over 90% of participants had internet access and 94% had used telehealth. Approximately half of all participants agreed or strongly agreed that telehealth would be beneficial in the future because it better fit their schedules and they would not need to travel. However, approximately half of the participants also agreed or strongly agreed they would not be able to express themselves well and could not be examined when using telehealth. Indigenous participants were especially concerned about these issues when compared to other racial groups.

**Conclusions:**

This work describes findings from a mixed methods community-engaged research study about telehealth, including perceived benefits and concerns. Although participants enjoyed the benefits of telehealth (eg, not having to travel and easier scheduling), they also had concerns (eg, not being able to express themselves well and not having a physical exam) about telehealth. These sentiments were especially notable among the Indigenous population. Our work highlights the importance of fully understanding the impact of these novel health delivery modalities on the patient experience and actual or perceived quality of care received.

## Introduction

### Background

Much has been written about the impact of COVID-19 on health care delivery, especially the increased use of telehealth in expanded clinical situations, including primary care, mental health, prenatal care, substance use disorder, orthodontics, urology, inpatient care, emergency triage, and physical therapy [1-7].

Although various forms of telehealth existed for many years prior to the pandemic, especially in rural contexts, it was relatively underused or restricted to within health care systems [8-11]. However, within the first 3 months of the COVID-19 pandemic, telehealth policy makers loosened or lifted many legal and practical restrictions, and the use of telehealth increased by 766% while outpatient visits dropped by over 80% in some institutions [12-14]. In the early stages of the COVID-19 pandemic, telehealth was primarily adopted to reduce viral transmission. As a mechanism to preserve personal protective equipment, even inpatients who required consultations with subspecialized teams or interactions with multidisciplinary members of the health care team sometimes received their consults via video conference [15,16].

### Telehealth Versus Telemedicine

The Federal Communications Commission defines telemedicine as medical services provided with the support of telecommunications technologies, such as diagnostic testing or monitoring a patient’s posttreatment progress. Telehealth includes a broader scope of clinical and nonclinical remote health care services—often provided by nurses—such as patient education, help with medication adherence, and troubleshooting health issues [17]. The World Health Organization has defined telehealth as being health care services provided by any health care professional and telemedicine as being services provided by a physician; however, the two terms are often used interchangeably [18].

Some observers predict that telemedicine and telehealth should be the delivery paradigm of the future because of their potential to equalize access to health care. Others have highlighted the potential of telemedicine and telehealth to exacerbate current inequality and inequity in health and access to health care [19] by raising barriers for those who do not have internet access, are disadvantaged from a socioeconomic perspective, are less digitally literate, or face language barriers [20-24]. Much attention has also been paid to privacy, data security, and connectivity concerns, which can threaten the rollout and sustainability of telehealth [25,26]. Finally, cultural and relational barriers may hamper the effectiveness of telehealth. Some individuals have reported that a remote discussion with a health care provider does not constitute an effective health care interaction [27].

Substantial literature has been published during the pandemic documenting the increased uptake and use of telehealth, but there has been less research focus on garnering the views of telehealth users [28,29]. Although some studies have examined the concerns of health care professionals, few have captured the experiences and perceptions of the public about these adaptations and the use of telehealth, including concerns [30-35]. Furthermore, studies capturing the views of diverse and underresourced communities about telehealth have been lacking.

This study is part of a larger community-engaged research (CEnR) study conducted over a 9-month period that used mixed methods to assess the impact of COVID-19 on marginalized communities across diverse geographic regions. CEnR has been defined by the Centers for Disease Control and Prevention as “the process of working collaboratively with and through groups of people affiliated by geographic proximity, special interest, or similar situations to address issues affecting the wellbeing of those people” [36,37]. CEnR can support the translation of scientific discovery into a reduction in heath disparities as it seeks to understand stakeholder and community needs. CEnR can foster benefits such as influencing public health initiatives and improved health outcomes [38,39].

This work describes differences in perceptions and experiences of telehealth during COVID-19 across vulnerable and underresourced communities, including apprehensions, perceived quality of care received, and potential advantages.

## Methods

### Study Setting and Design

We performed a multisite, multiphase, mixed methods study from January to November 2021 in three geographic regions of the United States: the southwest, southeast, and midwest [40]. We used CEnR methods to explore the needs, assets, and challenges of surrounding communities to understand the impact of COVID-19. Details of the methods are described elsewhere [41] but are briefly outlined below.

This project is part of a broader effort toward exploring several issues about the impact of COVID-19 on historically marginalized communities. We here focus on the content that informs considerations around telehealth. The study employed quantitative and qualitative methods in three phases. Phase 1 involved qualitative key informant interviews, phase 2 involved focus groups (FGs), and phase 3 employed an anonymous online survey [42]. As stated by Kelle et al [43], mixed methods research can support “mutual validation of data” as well as provide a “more coherent and complete” picture than a single research strategy alone.

### Ethical Considerations

The qualitative phases of the study were approved as minimal risk by the Mayo Clinic Institutional Review Board (21-001802 and 21-002163). The quantitative phase was hosted by an external survey research company. As verified by Research Compliance, Mayo Clinic had no participant contact, was therefore not engaged in human subjects’ research, and this phase was not eligible for Institutional Review Board review.

All data analysis was conducted by our study team. All procedures followed were in accordance with the ethical standards of the responsible committee on human experimentation and with the Helsinki Declaration of 1975, as revised in 2000.

During FGs, the moderator began the activity by outlining the elements of informed consent that had been shared prior to scheduling with participants and they were asked to confirm consent verbally. Participants received a US $50 financial remuneration for their time. As with most surveys, respondents are assumed to have provided consent if they complete the survey. Those who completed the survey received US $10 digital financial remuneration. All data from the survey and FGs were deidentified and stored on password-protected computers or in locked cabinets to which only the study team had access.

We here report the phase 2 perspectives of FG participants about their experience with telehealth and the phase 3 survey participant responses about telehealth.

### FGs and Surveys

#### FG Recruitment and Data Collection

The broader multidisciplinary study team developed the moderator guide, which included questions about the impact of COVID-19 on access and use of health care. The moderator’s guide covered the following COVID impact topics: future hopes, personal, medical, worries, vaccines, household, mental health, and community impacts. We disseminated flyers in Spanish and English through social media and community organizations and convened FGs in all three regions. The purpose of the study, risks and benefits, and the option to decline any question and/or withdraw at any time were outlined to participants before scheduling. Participants were placed in FGs with others who represented similar demographic, residential, or social communities. We also collaborated with local Departments of Health who were conducting FGs about the impact of COVID-19 on communities and were able to negotiate data sharing. Most FGs were conducted via online videoconferencing (participants could also join by calling in on a mobile phone without requiring internet access); some were conducted in person. FGs were scheduled for 1 hour. Two FGs were conducted in Spanish with bilingual moderators.

#### Survey Instrument and Sampling

We developed an electronic survey with the input of community and scientific leaders as well as other key stakeholders. The survey questions about telehealth were adapted from questions developed to evaluate the attitudes of patients living with HIV about telehealth in lieu of face-to-face visits [44]. They included questions about internet access, use of telehealth since the start of the pandemic, likelihood of using telehealth in the future, as well as perceived benefits of telehealth (fit schedule, no need to travel) and concerns with telehealth (doctor could not examine me, cannot express myself, information not safe, excess data usage) (see Multimedia Appendix 1 for full item wording of the survey questions). The survey was pilot-tested extensively, and we solicited comments and suggestions from Community Advisory Boards, existing panels of health equity, and community engagement researchers across the institution as well as volunteers from the community networks of those researchers [45]. The survey was also translated into Spanish by a licensed medical translator and tested by bilingual English/Spanish speakers.

The survey was distributed through existing networks of community partners and organizations using social media and email lists for 8 weeks in the fall of 2021. A professional research firm hosted, accrued, and deidentified the data.

### Analysis

#### FG Qualitative Data Analysis

We digitally recorded the FGs, and they were transcribed by a Health Insurance Portability and Accountability Act–compliant professional firm and deidentified before analysis. Data from FGs conducted by our team and those conducted by others were stored and analyzed in parallel using identical coding structures, but were not combined into one data set. We used the framework analytic approach to code our data [46] in which the verbatim content of FG transcripts is summarized in a matrix. In this matrix, deductive themes from group moderation guides create the columns and individual FGs create the rows. Inductive—emerging—themes may add additional columns. Transcript page numbers are noted with paraphrased content within the relevant cells. Two trained coders collaborated on all phases of analysis, including initial content paraphrasing, indexing to codebook development, and illustrative quote retrieval.

#### Survey Data Analysis

Statistical analyses were performed using SAS Software 9.04 (SAS Institute Inc, Cary, NC). Categorical variables are reported as counts with percentages and were analyzed with the Pearson *χ*^2^ test, Fisher exact test, and general linear models with Tukey options to test simultaneous differences between percentage means where appropriate. Continuous variables are reported as medians with IQR and analyzed with the Wilcoxon rank-sum test. A 2-sided *P* value of less than .05 was considered statistically significant. When looking at the difference between means with the Tukey honest-significant test, we tested if the difference between means is equal to zero. This may occasionally result in a significant difference between two groups even when the confidence intervals for those two groups overlap.

## Results

### FG Participants’ Baseline Characteristics

In total, data were analyzed for 47 FGs conducted during 2021 by our team and colleagues at Arizona State University. FGs were demographically and geographically discrete, and included separate groups who were uninsured and underinsured, those experiencing homelessness, LGBTQI, Latinx women and men, parents of young children, cancer survivors, Black men and women, Indigenous Americans, immigrants and Spanish-speaking adults, veterans, and older Asian Americans (see Multimedia Appendix 2).

### Survey Respondents’ Baseline Characteristics

The completed survey data set included 3447 English-language responses and 146 Spanish-language responses. Responses are proportionate to the geographic population of the area surveyed. Due to the mode of dissemination, we cannot calculate a response rate (see Table 1 for respondent characteristics). We conducted site-specific analysis as well as analysis across different demographic groups that included all sites. Of note, the subset identifying their primary identity as Asian was too small to analyze and has been excluded from this paper but included in other work.

**Table 1:** Survey respondent characteristics (N=3593).

Characteristics			Respondents, n (%)
**Location**			
	Arizona	2296 (63.9)	
	Florida	427 (11.9)	
	Midwest	870 (24.2)	
**Age (years)**			
	18-29	739 (20.7)	
	30-39	2068 (57.9)	
	40-49	700 (19.6)	
	50-75	68 (1.9)	
**Sex at birth**			
	Female	1164 (67.5)	
	Male	2420 (32.5)	
	Woman	2292 (65.0)	
	Man	1193 (33.9)	
	Nonbinary	22 (0.6)	
Prefer not to say gender			17 (0.5)
**Ethnicity/race**			
	Black Hispanic	342 (9.5)	
	Black non-Hispanic	427 (11.9)	
	Indigenous	269 (7.5)	
	White Hispanic	707 (19.7)	
	White non-Hispanic	1725 (48.0)	
	Other^a^	123 (3.4)	
**Education**			
	High school diploma or less	835 (23.4)	
	Some college or more	2733 (76.6)	

^a^Includes Asian.

### Internet Access

Internet access was high across all sites with over 90% of participants reporting access. Access was the highest among southwest participants, with 98% reporting internet access. Black Hispanics, Indigenous, and white non-Hispanics had lower rates of internet access than other racial and ethnic groups such as Black non-Hispanic and white Hispanic. No significant differences were detected across groups defined by age, sex, gender identity, or education (see Multimedia Appendix 3).

### Use of Telehealth to Date

A total of 3260 participants answered the question about use of telehealth since the start of the pandemic. Across all sites, any use of telehealth during this time was high with 94% (95% CI 93.2%-94.7%) overall using telehealth at some stage. The proportions (95% CIs) for telehealth use by subgroup are presented in Table 2. All three locations showed statistically significant different usage of telehealth. Florida had the lowest rate, with 88% (95% CI 85.9%-90.6%) having used telehealth. In Arizona, 94% (95% CI 92.3%-94.3%) and in the midwest 97% (95% CI 94.9%-98.2%) had used telehealth since the COVID-19 pandemic started. Black Hispanics had the highest use of telehealth at 97.7% (95% CI 95.1%-99.9%), which was significantly greater than the use of white non-Hispanics at 94.4% (95% CI 92.3%-94.5%). Among those aged 30-39 years, 94.8% (95% CI 93.7%-95.8%) used telehealth, similar to the rate of those aged 40-49 years at 94.3% (95% CI 92.5%-96.1%). This was significantly more than that of participants aged 18-29 years (89.6%, 95% CI 87.8%-91.4%) but was not significantly higher than the use rate of those aged 50-75 years (88.2%, 95% CI 82.4%-94.1%). There were no significant differences in use of telehealth based on education, sex, or gender identity.

**Table 2:** Use of telehealth to date according to participant characteristics.

Characteristics			Use telehealth, % (±95% CI)		Tukey groupings (difference between means)^a^		*P* value	
**Region**								<.001
	Midwest	96.1 (1.6)		A				
	Florida	88.3 (2.3)		B				
	Arizona	93.3 (0.9)		C				
**Age (years)**								<.001
	18-29	89.6 (1.8)		A				
	30-39	94.8 (1.1)		B				
	40-49	94.3 (1.8)		B				
	50-75	88.2 (5.8)		A, B				
**Sex at birth**								.05
	Female	92.4 (1.4)		A				
	Male	94.0 (1.0)		A				
**Ethnicity/race**								<.001
	Black Hispanic	97.7 (2.5)		A				
	Black non-Hispanic	94.4 (2.3)		A, B				
	Indigenous	93.3 (2.8)		A, B				
	White Hispanic	93.5 (1.8)		A, B				
	White non-Hispanic	93.4 (1.1)		B				

^a^Groups with different letters have a mean difference significantly different than 0 at the *P*=.05 level. No significant differences were detected across groups defined by sex, gender identity, or education.

### Perceived Benefits of Telehealth

Survey questions about the perceived benefits of telehealth included those related to telehealth fitting into people’s schedule better and not needing to travel.

The proportions (95% CIs) for perceived telehealth benefits by subgroup are presented in Table 3. Over half of all respondents (56.1%, 95% CI 54.5%-57.8%) agreed or strongly agreed that telehealth fit their schedule better than in-person visits. Indigenous respondents were significantly more likely to feel this way than any other groups except non-Hispanic white individuals, at 65.8% (95% CI 59.9%-71.7%) agreement. Those with moderate levels of education (59.0%, 95% CI 57.1%-60.8%) were significantly more likely than those with lower levels (48.5%, 95% CI 45.2%-51.9%) to report that telehealth fit their schedule better (see Table 3).

Those with moderate levels of education were significantly more likely to agree that telehealth would reduce the need to travel than those with lower levels of education (49.0%, 95% CI 47.2%-50.9% vs 38.8%, 95% CI 35.4%-42.2%). Additionally, the Indigenous population was significantly more likely to agree that not traveling was a benefit than any other group, at 64.3% (95% CI 58.4%-70.2%). There was a marginal age difference, with those aged 50-75 years being least likely to agree (33.8%, 95% CI 22.0%-45.7%). This was significantly lower than that for participants aged 40-49 years (50.7%, 47.0%-54.4%), but not for the other age groups (see Table 3).

**Table 3:** Perceived benefits of telehealth.

Characteristics		Fit schedule better					Would not need to travel		
		Agree, % (± 95% CI)	Tukey groupings (difference between means)^a^	*P* value		Agree, % (±95% CI)		Tukey groupings (difference between means)^a^	*P* value
**Region**				.004					.05
	Midwest	57.2 (3.3)	A			43.1 (3.3)		A	
	Florida	48.9 (4.7)	B			48.7 (4.7)		A	
	Arizona	57.5 (2.0)	A			47.6 (2.0)		A	
**Age (years)**				<.001					.01
	18-29	50.2 (3.6)	A			44.2 (3.6)		A, B	
	30-39	58.1 (2.1)	B			46.6 (2.1)		A, B	
	40-49	59.6 (3.7)	B			50.7 (3.7)		A	
	50-75	45.6 (11.8)	A, B			33.8 (11.8)		B	
**Ethnicity/race**				.002					<.001
	Black Hispanic	50.6 (5.3)	A			43.6 (5.3)		A	
	Black non-Hispanic	55.0 (4.7)	A			40.5 (4.7)		A	
	Indigenous	65.8 (5.9)	B			64.3 (5.9)		B	
	White Hispanic	53.6 (3.7)	A			46.7 (3.7)		A	
	White non-Hispanic	57.0 (2.3)	B			44.8 (2.3)		A	
**Education**				<.001					<.001
	High school or less	48.5 (3.4)	A			38.8 (3.4)		A	
	Some college or more	59.0 (1.9)	B			49.0 (1.9)		B	

^a^Groups with different letters have a mean difference significantly different than 0 at the *P*=.05 level. No significant differences were detected across groups defined by sex or gender identity.

### Perceived Concerns With Telehealth

Survey questions about the perceived concerns of telehealth included those related to not being examined properly and not being able to express oneself.

The proportions (95% CIs) for perceived telehealth concerns by subgroup are presented in Table 4. Overall, approximately half of all respondents (48.2%, 95% CI 46.5%- 49.8%) felt that telehealth would hinder the ability of the provider to examine them well. This pattern was consistent across all demographic groups with some notable differences. Those aged 30-39 years and 40-49 years were the most concerned about this issue at 49.4% (95% CI 47.2%-51.5%) and 53.4% (95% CI 49.7%- 57.1%), respectively, which was significantly higher than the rate of agreement for those aged 18-29 years at 43.4% (95% CI 39.8%-47.0%). Those with moderate levels of education were significantly more likely to be concerned about this issue (49.9%, 95% CI 48.1%-51.8%) than those with lower levels of education (38.8%, 95% CI 35.4%-42.2%). Indigenous respondents were statistically the most concerned about lack of sufficient examination through telehealth, with 63.9% (95% CI 58.2%-69.7%) agreeing or strongly agreeing that “the doctor would not be able to examine me well.” In all other racial and ethnic groups, only about half of all respondents agreed or strongly agreed with the statement. No significant differences were detected across groups defined by sex or gender identity (see Table 4).

Overall, 45.2% (95% CI 43.5%-46.8%) of participants agreed or strongly agreed that they “would not be able to express themselves well.” The participants in the southeast differed from those of other sites, with only 38.4% (95% CI 33.7%-43.1%) concerned about the issue of expressing themselves. Indigenous respondents had more concerns than other demographic groups, with 60.2% (95% CI 54.3%-66.1%) agreeing or strongly agreeing that they “would not be able to express themselves well.” No statistically significant differences between age groups, sex, gender, or education level were noted (see Table 4).

**Table 4:** Perceived concerns with telehealth.

Characteristics		Could not be examined				Could not express myself		
		Agree, % (±95% CI)	Tukey groupings (difference between means)^a^	*P* value	Agree, % (±95% CI)		Tukey groupings (difference between means)^a^	*P* value
**Region**				.06				.001
	Midwest	49.0 (3.3)	A, B		49.2 (3.3)		A	
	Florida	43.6 (4.7)	B		38.4 (4.7)		B	
	Arizona	49.7 (2.0)	A		45.8 (2.0)		A	
**Age (years)**				.002				.30
	18-29	43.4 (3.6)	A		43.0 (3.6)		A	
	30-39	49.4 (2.2)	B		47.1 (2.1)		A	
	40-49	53.4 (3.7)	B		45.3 (3.7)		A	
	50-75	47.1 (11.9)	A, B		45.6 (11.8)		A	
**Ethnicity/race**				<.001				<.001
	Black Hispanic	44.7 (5.3)	A		40.0 (5.2)		A	
	Black non-Hispanic	41.9 (4.7)	A		39.3 (4.7)		A	
	Indigenous	63.9 (5.9)	B		60.2 (5.9)		B	
	White Hispanic	45.8 (3.7)	A		43.6 (3.7)		A	
	White non-Hispanic	48.9 (2.3)	A		46.0 (2.3)		A	
**Education**				.02				.66
	High school or less	45.3 (3.4)	A		45.1 (3.4)		A	
	Some college or more	49.9 (1.9)	B		46.0 (1.9)		A	

^a^Groups with different letters have a mean difference significantly different than 0 at the *P*=.05 level. No significant differences were detected across groups defined by sex or gender identity.

### FG Data

FGs discussed the broad issue of the impact of COVID-19 on medical care. Telehealth was one of several impactful topics within this realm. Some FG participants supported the concept of the convenience of telehealth, reflecting the findings from the survey data. These views were found in different geographic locations in which FGs were conducted. Participants found telehealth easier, convenient, and helpful, as it avoided the need to travel and park at a clinic, facilitated health care discussions, and was less disruptive than going in person. Textbox 1 provides representative quotes from the FG discussions highlighting these benefits.

**Representative quotes from focus group discussions on the benefits of telehealth during the COVID-19 pandemic.**

“Everything I have done is virtual which honestly for my lifestyle is more convenient for me.” [Florida, Black millennials/Black women focus group]

“Fortunately enough, I’ve had…help and virtual doctors where I can tell them my symptoms …, and then they’ll prescribe me medicine, and I can just pick it up. That’s been really helpful.” [Arizona, LGBTQ+ focus group]

“…we now have access to telehealth. …and I think that, in the end, it’s been positive for those people who don’t have access to go to a clinic personally.” [Florida, Spanish-speaking populations focus group]

“We actually prefer virtual, just so then we don’t actually have to get over there and drive and pay for parking and go back.” [Midwest, pregnancy focus group]

“My doctors are in [a different location] and it makes it easier for us to talk to them virtually.” [Arizona, Gila Bend, Maricopa County focus group]

“There’s a discretionary process to see should you even be in-person, or can we do this virtually. I had an ear infection a couple of months ago... (they said) ‘we'll put you on with a nurse and you can describe all your symptoms. If it’s actually an ear infection, we’ll put in a prescription for you and we won’t make you come in’.” [Florida, Black millennials/Black women focus group]

However, as in the survey findings, several FG participants had concerns about telehealth. These views included worries about not being assessed adequately, lack of trust in the sufficiency of telehealth, feeling uncomfortable, and sometimes just wanting to see a clinician in person. These views were found across all geographic locations in which FGs were conducted. Textbox 2 provides representative quotes about these concerns.

**Representative quotes from focus group discussions on the concerns of telehealth during the COVID-19 pandemic.**

“During COVID, everything was via video call. … I did not feel comfortable about it because I wanted to see …the doctor personally.” [Florida, Spanish-speaking populations focus group]

“It’s awkward. I prefer being touched and having them look at my eyes to see how I am. They can’t do that over the internet.” [Florida, unhoused people focus group]

“Well, it’s hard to diagnose anybody or see anybody, how they’re really doin’ or whatever when you don’t see them physically. You don’t really see what’s goin’ on.” [Arizona, Guadalupe, Maricopa County focus group]

“I feel like remote visits to the doctor are fairly worthless. *…* You’re just telling them your symptoms. They can’t look at you and assess you in person. I feel like I just paid $100 for nothing…” [Arizona, parents with young children focus group]

“Theoretically, you have everything you need there, but you don’t. You're missing a third dimension.” [Arizona, veterans focus group]

“You just don’t feel satisfaction (with video calls). We had our annual check-up, for the year, 2020, using telehealth. I know my wife didn’t feel that a thorough evaluation of her health condition with the general practitioner got done. Obviously, I feel somewhat similar too.” [Arizona, Asian American 65+ focus group]

Furthermore, some FG participants articulated a concern with not being listened to or understood, including themes of minimization of symptoms, potential for medical errors, and lack of high-quality care.

"It didn’t feel like they were actually hearin’ or seein’ like they would in person. It’s like, “Okay, take an aspirin and call me in the mornin’. See how you feel.” That was my opinion of it, and I didn’t feel I was gettin’ the personal care that I needed from my doctors."

Florida, unhoused people FG

"you get a lot of different opinions. It’s virtual. It’s not real in person. You get misdiagnosed or told different things from different doctors."

Arizona, LGBTQ+ FG

Notably, sometimes within the same FG, divergent opinions and perceptions of telehealth were voiced. For example, participants in the LGBTQ+ and the Spanish-speaking FGs expressed both positive and negative perceived views of telehealth. Those who were unhoused articulated negative views about telehealth overall.

## Discussion

### Principal Findings

This CEnR, mixed methods study used a community survey and FGs to collect data from diverse and underserved populations across three regions of the United States about the use and perceptions of telehealth from January to November 2021 of the COVID-19 pandemic. Although telehealth was widely used by all demographic groups and in all states, perceived benefits and concerns differed. Despite frequently voiced apprehension in the news media about connectivity and its impact on telehealth, over 90% of our participants had internet access.

What was most striking from the survey data findings was the coexisting perceived benefits and concerns among Indigenous respondents. Indigenous respondents, like other ethnic groups, enjoyed the convenience of telehealth as they did not need to travel and it fit in their schedule; however, they also had greater concerns than other groups about telehealth, including the ability of their clinicians to examine them properly and for them to be able to express themselves to their clinicians. Evidence from studies conducted prepandemic suggested that lack of physical examination has been a consistent concern for patients. Guidelines to help determine the appropriateness of a virtual visit as well as guidance for conducting virtual physical examinations have been developed as a result [47,48].

TechQuity is a recently developed term that incorporates an antiracism and proequity ethos to the use of technology in health care. While telehealth is a small part of the increasing use of technology within the health care realm, we should be mindful that telehealth does not exacerbate structural racism and inequities and take note of these findings [49]. Other potential barriers to the use of telehealth not assessed in our study relate to digital literacy and comfort with technology, which may be worse among older adults. This group may benefit substantially from avoiding in-person visits owing to their susceptibility to contagion and mobility challenges [50,51]. However, they faced challenges with online scheduling of vaccination appointments and may require assistance to use telehealth, which may not always be available [52,53]. Language barriers and poor health literacy may also be obstacles to the use of telehealth [54,55]. Just as the above factors substantially impacted vaccine uptake due to difficulties in accessing patient portal messages and online scheduling, it is likely that a telehealth visit without sufficient support from a caregiver or family member would not meet patient needs sufficiently and detract from the patient experience [53,56].

Since the previously ubiquitous reimbursement and licensure hurdles that hampered adoption have been largely overcome during the pandemic, telehealth is appealing to institutions as a mechanism to efficiently deliver some forms of health care [10]. The exponential increase in the use of telehealth during the pandemic has encouraged health care institutions to maintain telehealth as a permanent modality for delivering certain types of care [57-59]. While telehealth has some advantages for convenience as highlighted in our findings, concerns remain and warrant further exploration, particularly as they relate to the patient experience.

### Study Strengths

We leveraged CEnR methodology as an important tool to understand and address equity issues associated with telehealth in our communities [37,38]. The study utilized a mixed methods approach within a limited time frame across multiple states and sites safely during the pandemic.

We disseminated a community-wide electronic survey among diverse and underserved populations using our community networks, partnerships, and organizational connections, utilizing social media to reach those not usually surveyed by academic institutions. We did not use a convenience sample of empaneled patients. The survey was translated into Spanish using a licensed medical translator and it was pilot-tested by bilingual English/Spanish speakers. Participants needed only a mobile phone to participate in both the surveys and FGs. We combined our quantitative survey methods with qualitative research approaches using FGs, providing us with a deeper contextual understanding of the data [40]. We had a multidisciplinary study team who had expertise in survey methods, qualitative methods, and mixed methods, ensuring all phases of the research were conducted in a scientifically robust manner [60].

Another strength of this study is the use of the framework method to analyze the FG data. This approach has several advantages over other qualitative analytic methods. It can be deployed in multidisciplinary research teams where some members have limited qualitative experience but can still engage in sense-making under the guidance of an experienced qualitative researcher. Furthermore, the framework analysis supported a structure for cross-disciplinary analysis of matrices we developed over time as well as a robust audit trail for our study team [46].

### Study Limitations

As with all surveys, response bias is a potential concern. It is possible that those with strong opinions are more likely to respond. We are unable to calculate a response rate for the survey due to the social media mode of dissemination, and while this was assessed to be the most successful mode for capturing diverse and underserved community voices, people who do not use social media may have been less likely to be aware of the study. Nonusers of social media may also be less familiar with digital technology; thus, these findings may underestimate some of the potential concerns. We conducted most FGs through Zoom technology, which likely supported engagement among most participants and was less burdensome than traveling for an in-person FG, but it is possible that this deterred some from participating or fully engaging. Even with broad-reaching recruitment strategies, we received few survey responses from those identifying as Asian. Our findings elucidate important technological considerations about telehealth, including internet access, but also other less explored worries that patients may have about whether telehealth is as effective as in-person clinic visits.

It is likely that health care systems will have differing priorities to patients when decisions about telehealth access and utilization are discussed [61-63]. Health care systems that invested time, staff, and other resources into supporting telehealth have been able to overcome barriers such as reimbursement and now hope to promote telehealth in many health care settings as a viable return on investment [63]. As health care institutions roll out and sustain telehealth, considerations need to include not only internet access and connectivity but also ease with use of telehealth based on education, language, technology savvy and health literacy, and the patient experience.

### Conclusion

This manuscript describes findings from a mixed methods CEnR study about telehealth, including perceived benefits and concerns. As well as being thoughtful about the logistical elements of implementing new health care delivery modalities and processes, our work also highlights the imperative to understand the impact of these novel modalities on the patient experience and actual or perceived quality of care received. Although telehealth is popular among health care systems, overall, patients have concerns that include not being able to express themselves and not having a physical exam.

## Appendix

Multimedia Appendix 1 Survey questions related to telehealth.

Multimedia Appendix 2 Focus group participant baseline characteristics.

Multimedia Appendix 3 Internet access according to demographic characteristics of survey participants.

## Abbreviations

CEnR: community-engaged research
FG: focus group
