# Supplementary material for: Perceptions and Use of Telehealth Among Diverse Communities: Multisite Community-Engaged Mixed Methods Study
Source: J Med Internet Res. 2023 Mar 28;25:e44242. doi: 10.2196/44242 (PMC10057900; doi:10.2196/44242)
Supplement: Multimedia Appendix 1 [file jmir_v25i1e44242_app1.docx]

Multimedia Appendix 1: Survey questions related to telehealth:

Q8.1 We are interested in your experiences with accessing health care remotely during the COVID-19 pandemic and your opinions about telehealth.

Q8.2 Do you have access to the internet in your home?

- Yes (1)
- No (2)

Q8.3 **Since the start of the COVID-19 pandemic**, have you used live video calls (like Skype, Zoom, Facetime, live chat…) to see and talk to your doctor instead of going
 to clinic appointments?

- No (1)
- Yes, some of the time (2)
- Yes, about half the time (3)
- Yes, most of the time (4)
- Yes, every time (5)

Q8.4 If you could use live video calls (like Skype, Zoom, Facetime, live chat…) to see and talk to your doctor instead of coming to clinic appointments, how likely would you use it?

- Very unlikely (1)
- Somewhat unlikely (2)
- Neither likely nor unlikely (3)
- Somewhat likely (4)
- Very likely (5)

Q8.5 For these questions, think about how telehealth would affect your future health care.

|  | Strongly disagree (1) | Disagree (2) | Neither Agree nor Disagree (3) | Agree (4) | Strongly Agree (5) |
| --- | --- | --- | --- | --- | --- |
| Live video calls with my doctor (telehealth) would help me because it would fit my schedule better than in-person visits (1) |  |  |  |  |  |
| Live video calls with my doctor (telehealth) would help me because I would not have to travel to a clinic (2) |  |  |  |  |  |
| In live video calls with my doctor, my doctor would not be able to examine me well (3) |  |  |  |  |  |
| In live video calls with my doctor, my personal information would not be safe (4) |  |  |  |  |  |
| In live video calls with my doctor, I would not be able to express myself very well (5) |  |  |  |  |  |
| In live video calls with my doctor, I would use too much data on my phone service or internet (6) |  |  |  |  |  |
