# Supplementary material for: Perceptions and Use of Telehealth Among Diverse Communities: Multisite Community-Engaged Mixed Methods Study
Source: J Med Internet Res. 2023 Mar 28;25:e44242. doi: 10.2196/44242 (PMC10057900; doi:10.2196/44242)
Supplement: Multimedia Appendix 2 [file jmir_v25i1e44242_app2.docx]

Multimedia Appendix 2: Focus Group Participant Characteristics

| Site | Groups | Group Description |
| --- | --- | --- |
| **Maricopa County (Phoenix) area, Arizona** | 31* | Geography-based: NE, SE, NW, SW, Central, South Central  Attribute-based: Latino men, Black/AA women, Parents w/kids at home, Asian Americans, LGBT+, Indigenous |
| **Duval County (Jacksonville) area, Florida** | 7 | Attribute-based: LGBT+, Black/AA women, Recently pregnant, Latina women, Cancer survivors |
| **Olmsted County (Rochester) area,**  **Minnesota; and**  **La Crosse County (La Crosse) area, Wisconsin** | 9 | Attribute-based: Latino men, Latina women, Young adults, Low income, Immigrant/refugee, LGBT+, Recently pregnant, Cancer survivors |
