# Supplementary material for: Perceptions and Use of Telehealth Among Diverse Communities: Multisite Community-Engaged Mixed Methods Study
Source: J Med Internet Res. 2023 Mar 28;25:e44242. doi: 10.2196/44242 (PMC10057900; doi:10.2196/44242)
Supplement: Multimedia Appendix 3 [file jmir_v25i1e44242_app3.docx]

Multimedia Appendix 3: Internet Access

* Groups with different letters have a mean difference significantly different than zero at the *P*=.05 level. No significant differences were detected across groups defined by age, sex, gender identity, or education.
